# Rapid Tranquilisation Use in Working-Age Adult Inpatients

**DOI:** 10.1192/bjo.2022.409

**Published:** 2022-06-20

**Authors:** James O'Neill

**Affiliations:** Leeds and York Partnership Foundation Trust, Leeds, United Kingdom. University of Leeds, Leeds, United Kingdom

## Abstract

**Aims:**

This study aimed to review if clinicians varied significantly in choosing rapid tranquillisation agents when using consistent clinical guidelines, analysing the rationale behind decision-making. It also aimed to assess confidence across varying grades and clinical experience, and to evaluate efficacy of current trust guidelines. We hypothesized that less experienced clinicians would be less willing to prescribe antipsychotics for rapid tranquillisation, and that current guidelines did not allow for consistent and uniform prescribing.

**Methods:**

A qualitative survey was distributed to 165 clinicians within one mental health trust, including core psychiatry trainees, trust-grade doctors, higher trainees, staff-grade doctors & working-age adult consultants. This survey included a fictional but commonly occurring scenario which clinicians responded to with the aid of current trust guidance. Respondents were then asked to justify their choice and to rank their confidence in prescribing rapid tranquillisation, along with rating how useful the guideline was in aiding their decision. Thirty-six participants responded to this survey, with a response rate of around 22%. There was even representation across clinical grades.

**Results:**

Clinicians of all grades were equally willing to prescribe antipsychotic agents for rapid tranquillisation. Higher psychiatric trainees reported greatest self-confidence when prescribing tranquillisation, with consultants surprisingly lower in confidence. Intramuscular olanzapine was most favourable, but significant variability was observed in suggested management between clinicians. Main themes for suggested amendments to the guideline included clarity, when to use the various options, further specification on dosage ranges and options for specific instances, such as if a patient is antipsychotic naïve or there is minimal physical health information.

There was marked variability in choice of agent. The majority of clinicians felt that early commencement of antipsychotic was beneficial in acutely unwell patients, although the merits of initially assessing medication-free were also raised. Key themes for tranquillisation choice included a need for a prior electrocardiogram to prescribe intramuscular haloperidol, the potential lack of efficacy with aripiprazole, the risk of respiratory depression with concurrent olanzapine and lorazepam, and a surprisingly high proportion of respondents opting for combined use of haloperidol plus a further sedative.

**Conclusion:**

Less experienced clinicians were not found to lack confidence to prescribe antipsychotics for rapid tranquillisation. However, clinicians responding to the same clinical scenario using the same guideline resulted in marked variability in choosing rapid tranquillisation agents. This highlights a need for clearer guidelines and education on this matter to ensure a consistent treatment approach to tranquillising medication.

